# The protective effect of luteolin on cadmium induced liver intestinal toxicity in chicken by Gut-liver axis regulation

**DOI:** 10.1016/j.psj.2024.104242

**Published:** 2024-08-23

**Authors:** Hui Zou, Waseem Ali, Kai Deng, Yan Chen, Jian Sun, Tao Wang, Yonggang Ma, Zongping Liu

**Affiliations:** ⁎ College of Veterinary Medicine, Yangzhou University, Yangzhou, Jiangsu 225009, PR China; † Joint International Research Laboratory of Agriculture and Agri-Product Safety of the Ministry of Education of China, Yangzhou University, Yangzhou, Jiangsu 225009, PR China; ‡ Jiangsu Co-innovation Center for Prevention and Control of Important Animal Infectious Diseases and Zoonoses, Yangzhou, Jiangsu 225009, China

**Keywords:** cadmium, luteolin, gut-liver axis, intestinal microbiota, correlation analysis

## Abstract

Environmental pollution poses a significant challenge to the poultry industry, leading to substantial losses and adverse effects on the health, production, and performance of avian species. In recent years, there has been growing interest in exploring natural compounds with potential protective effects against cadmium (**Cd**)-induced toxicity. Luteolin (**LUT**), a flavonoid found in various plants, has been studied for its antioxidant, anti-inflammatory, and cytoprotective properties. In this study, Su green shell grass chickens were divided into 4 groups: control, LUT (150 mg LUT), Cd (100 mg CdCl2), and Cd + LUT (100 mg CdCl2 + 150 mg LUT) groups for 1 month, respectively. The present study revealed that LUT maintained the morphology and functional activity of the liver and intestine. LUT alleviated Cd-induced impairment in the liver and intestinal biochemical indicators, suppressed Cd-induced liver fibrosis, mitigated liver and intestinal tissue damage. Additionally, LUT reduced oxidative stress and regulated the Cd-induced impairment in trace elements of the liver and intestine. Furthermore, LUT reduced Cd-induced liver inflammation, restored Cd-induced intestinal barrier function, and normalized Cd-induced serum proteins, including changes in the content of glutamyltranspeptidase. Moreover, LUT maintained Cd-induced disruption of gut microbiota and alleviated bacterial dysbiosis. Overall, these findings suggest that LUT holds promise as a potential therapeutic agent for mitigating the adverse effects of Cd-induced toxicity in poultry, by preserving liver and intestinal health, reducing oxidative stress, inflammation, and restoring gut microbiota balance.

## INTRODUCTION

Cadmium (**Cd**) is a highly toxic substance extensively distributed and persists in the environment due to ever-increasing urbanization, industrial revolution, and human activities (Wani et al., 2020). The situation has severely threatened the health of humans and animals (Genchi et al., 2020; Huang et al., 2019; Li et al., 2022; Ran et al., 2023). More importantly, once Cd is absorbed, it can accumulate in the kidney, liver, intestine, brain, and other organs, especially the liver and intestine, promotes the occurrence of a variety of chronic diseases (Arruebarrena et al., 2023; Souza-Arroyo et al., 2022; Vesey 2010). Several epidemiological studies on chronic liver diseases have consistently reported elevated levels of Cd in the blood of individuals with conditions such as liver fibrosis, nonalcoholic fatty liver disease (**NAFLD**) and cirrhosis. These findings indicate a positive correlation between Cd exposure and the development of chronic liver disease (Kolachi et al., 2012; Sun et al., 2024a). Cd-induces liver and intestine injury through multiple mechanisms, such as oxidative stress, autophagy, apoptosis, DNA damage, and metabolic disorders (Sun et al., 2024b; Zhang et al., 2022a). The intestinal barrier function is the most important function of the intestine in combating foreign pathogens. If the intestinal barrier function is disrupted, inflammatory bowel disease (**IBD**) quickly occurs, which in turn induces liver inflammation through the intestinal liver axis pathway (Pabst et al., 2023). For a considerable time, heavy metals have been implicated in damaging the digestive system. Recent studies have elucidated that exposure to Cd disrupts the structure of the intestinal tract, compromising its immune defense capacity and leading to inflammation (Xie et al., 2019). Oral administration of Cd has been found to induce intestinal injury and inflammation in rat (Ninkov et al., 2015). Most of the studies were investigated and showed Cd-induced liver and intestine injuries in mammals. However, understanding the pathogenesis of Cd-induced liver and intestine injury in avian species is still obscure.

Luteolin (**LUT**) is a natural flavonoid with multiple functions, widely present in plants such as pepper, wild chrysanthemum, purslane, honeysuckle, and perilla. LUT has various biological activities, such as anti-inflammatory, antioxidant, anti-allergic, uric acid lowering, anti-tumor, antibacterial, and antiviral activities. It is known that Cd can disrupt the oxidative antioxidant balance of liver cells, causing them to produce a large amount of ROS and causing oxidative stress in liver cells (Wang et al., 2018). LUT has strong antioxidant properties, it can alleviate Cd- induced liver damage in rats by alleviating oxidative stress and alleviating liver metabolic disorders (Shahzadi et al., 2023). Numerous studies have reported that LUT effectively mitigates pathological lesions induced by heavy metals in various organs through mechanisms involving the enhancement of antioxidant enzyme activities and the reversal of mitochondrial dysfunction (AL-Megrin et al., 2020; Domitrović et al., 2013; Tan et al., 2018). However, it is unclear whether LUT can alleviate cadmium-induced oxidative damage in the liver and intestine of chickens and the mechanisms involved in this process.

The gut-liver axis, a bidirectional communication pathway between the gastrointestinal tract and the liver, plays a crucial role in maintaining overall health and homeostasis (Albillos et al., 2020; Ding et al., 2020). Disruption of the gut-liver axis has been implicated in the pathogenesis of various liver diseases, including those induced by environmental toxins like Cd (Zhang et al., 2021). Understanding the interplay between the gut and liver in the context of Cd toxicity and LUT intervention could provide valuable insights into novel therapeutic strategies for mitigating Cd-induced liver and intestinal injury in chickens.

In this study, we aimed to investigate the protective effect of LUT on Cd-induced liver and intestinal toxicity in chickens by elucidating the mechanisms involved in the gut-liver axis regulation. Through comprehensive analysis of biochemical, histopathological, and molecular parameters, we aimed to shed light on the potential therapeutic role of LUT in alleviating Cd toxicity and restoring gut-liver axis homeostasis in chicken.

## MATERIALS AND METHODS

### Chemicals and Antibodies

The following antibodies and chemicals were used in this research. Hematoxylin and Eosin, and Sirius red staining (**SRS**) were supplied by Beijing Solaibao Biotechnology Co., Ltd. Enzyme-linked immunosorbent assay (**ELISA**) kit obtained from Bio Tek, USA. BA reagent kit, BCA protein concentration determination kit, CAT reagent kit, GSH reagent kit, LPS reagent kit, MDA reagent kit, RIPA cracking solution, T-AOC reagent kit, T-SOD reagent kit, Protease inhibitor mixture were supplied by Nanjing Jiancheng Biotechnology Co., Ltd. Bovine serum protein bought from Suzhou Xinsaimei Biotechnology Co., Ltd. Claudin-1 antibody, Claudin-4 antibody, GAPDH antibody, IL-18 antibody, IL-1 β antibody, NLRP3 antibody, Occludin antibody, TGF- β antibody and ZO-1 antibody were bought from Cell Signaling Technology, USA. Molecular weight standard for color pre stained proteins obtained from Shanghai Yisheng Biotechnology Co., Ltd. Hypersensitive ELC Chemiluminescence Kit was supplied by Suzhou Xinsaimei Biotechnology Co., Ltd. Second antibodies were supplied by Santa Cruz Biotechnology.

### Experimental Animals

A total of Forty Su poultry green shell grass chickens (1-day-old) were obtained from Yangzhou Xianglong Poultry Development Co., Ltd. using the principle of a single variable to keep all chickens under the same feeding conditions. All animal experiments were approved by the Animal Care and Use Committee of Yangzhou University and conducted in accordance with the Guidelines for the Care and Use of Experimental Animals.

### Experimental Design

Forty one-day-old chickens were employed in this study. Prior to the formal trial, all chickens were housed in a comfortable environment with unrestricted access to food and water. Following a 7-d adjustment period, the chickens were randomly divided into 4 groups of 10 each group. For 1 week, a dose of 150 mg of LUT was added per kilogram of body weight to the basic diet. Subsequently, following pretreatment with CdCl2 and LUT were administered via drinking water and the basic diet at concentrations of 100 mg/L and 150 mg/kg of body weight, respectively, for 1 month (Wang et al., 2024)." Control/untreated group (0 mg LUT + 0 mg CdCl2); LUT group (150 mg LUT); Cd group (100 mg CdCl2); and Cd + LUT group (100 mg CdCl2 + 150 mg LUT). After 1 month, the chickens were sacrificed with an overdose intravenous sodium pentobarbital injection (50 mg/kg), and targeted tissues (liver and intestine) were collected and stored at -80 °C for indicated experiments. This study was approved by the Animal Care and Use Committee of Yangzhou University (Approval Number: SYXK (Su) 2017-20044).

### Preparation of Reagents

Cadmium chloride solution: Weigh 100 mg of cadmium chloride powder (purity 99.99%, Sigma Aidrich), dissolve in 1 L of ultrapure water, and mix to volume for later use. LUT solution: Weigh the powder of LUT (purity 98%, Shanghai Yuanye Biotechnology Co., Ltd.) and dissolve it in an appropriate amount of ultrapure water. Prepare a 150 mg/kg LUT solution according to the weight of the chicken.

### Sample Collection

After a 12-h fasting period at the end of the 4-week period, the chickens were euthanized, and their weights were recorded. Blood samples were collected from the heart and centrifuged at 3,000 rpm for 10 min to obtain serum. Simultaneously, liver and intestinal tissues were promptly collected, weighed, and a portion of each was immersed in 10% neutral formalin for subsequent experiments.

### Evaluate Growth and Development Status

Calculated the water and food consumption and clinical manifestations of animals once a day, and measured their weight every 3 d for 30 d. After obtaining liver tissue, weigh it with an electronic balance and calculate organ coefficients. Organ coefficient (%) = (organ weight/body weight) x 100%. After obtaining intestinal tissue, measure the length of intestinal tissue using measuring paper.

### Measurement of Oxidative Stress Markers

According to the manufacturer's protocols, the activities of total antioxidant capacity (**T-AOC**), GSH, total superoxide dismutase (**T-SOD**), CAT and the content of hydrogen peroxide (**H_2_O_2_**) were determined. The corresponding test kits from Nanjing Jiancheng Biological Manufacture (Nanjing, China).

### Enzyme-Linked Immunosorbent Assay

The levels of inflammatory cytokines in spleen, including interleukins-12 (**IL-12**), IL-10, tumor necrosis factor (**TNF-α**, and **TGF-β**) were detected using the commercial ELISA kits. These kits were obtained from Shanghai Enzyme Linked Biotechnology Co., LTD (Shanghai, China). All procedures strictly follow the manufacturer's instructions.

### Detection of Trace Elements in Liver and Intestine

Trace elements were determined in digested solutions using Inductively Coupled Plasma-Optical Emission Spectrometry (Optima 2100, Perkin-Elmer, Shel ton, CT) (Osmond-McLeod et al., 2014). Liver and intestinal tissue samples from each group were placed in different labeled 5 mL EP tubes and cut with scissors. The samples were kept at a temperature of 80°C to dry out the water. The liver and intestinal tissue were then ground into a powder. The tissue was digested using a microwave digestion instrument: 200 mg of tissue powder and 4 mL of concentrated nitric acid were added into the digestion tubes, and tissue was microwave-digested at 150°C for 40 min. The digested tissue solution was then diluted with distilled water into a 10 mL solution and transferred to a new 15 mL centrifuge tube. The final elemental contents were analyzed using an atomic spectrometer.

### Hematoxylin Eosin Staining

HE staining was performed according to the procedures described by (Haseeb et al., 2019). Place the liver and intestinal tissues of chickens in 4% paraformaldehyde, fix them for about 12 h, rinse gently with distilled water, and let them stand overnight. After dehydration with different gradients of alcohol, and then tissue was embedded in paraffin wax, after that the tissue was sectioned (5 μm slice) using a microtome, then dewaxing of tissue slides was performed in different concentrations of alcohol, and finally staining with hematoxylin eosin solution, a light microscope was used to examine the stained sections. ImageJ software was used to analyze the villus length, crypt depth, and thickness of the 4 layers of intestinal hematoxylin eosin (**HE**) slices.

### Sirius Red Staining

Sirius Red staining was performed according to the procedure described by (Ali et al., 2024). The paraffin embedding steps are the same as above, resulting in 5 μm Slice the liver into sections, then remove wax, and finally stain with Sirius red. The Sirius red stained sections of the liver can be used to detect the degree of fibrosis in each group.

### Blood Biochemical Analyzer for Detecting Serum Biochemistry

After heart blood collection and centrifugation (3,000 r/min, 10 min), the supernatant was taken and the contents of inorganic phosphates, total cholesterol, triglycerides, serum bile acids, and blood calcium were measured using a fully automated blood biochemistry analyzer; Blood sugar; The activity of ALT, AST, and ALP.

### Western Blotting

The detailed methods of protein extraction and western blotting were described in our previous studies (Zhang, et al., 2022b). Liver and intestine protein samples were extracted using RIPA lysate (20101ES60, NCM, Suzhou, China), and protein concentrations were standardized using the BCA Protein Assay kit (P0009, Beyotime, Shanghai, China). Samples were added with SDS buffer and boiled for 8 to 15 min to denaturize the proteins. Protein samples were separated in 8% to 12% SDS-PAGE gels and subsequently transferred to PVDF membranes. Membranes were blocked with 5% nonfat milk or BSA solution and then incubated with primary antibodies overnight at 4°C. Subsequently, the membranes continued to be incubated with secondary antibody for 1 to 2 h at room temperature. Finally, western blot images were visualized by Tanon imaging system using enhanced chemiluminescence reagent (P10300, NCM, Suzhou, China). Protein expression was analyzed by Image J 1.42q software (NIH, Bethesda, MD) and normalized by β-actin.

### RT-qPCR Analysis

Relative mRNA expression levels of target genes in liver and intestinal tissue were detected by RT-qPCR. As previously mentioned, total RNA was extracted from liver and intestine, and cDNA was synthesized using Trizol reagent (Takara, China) and reverse transcription kit (Takara, China) according to the manuals, respectively (Wang et al., 2024). In this study, all details of primer sequences of genes were shown in (Table 1), and synthesized by Biotech Biotechnology (Shanghai, China) Co. The internal standard gene chosen was the β-actin gene. Importantly, the changes of relative mRNA expression levels of the target genes were calculated using the formula 2^−ΔΔCT^.

**Table 1:** List of primers.

Gene	Forward	Reverse
NLRP3	CCGCGTGTTGTCAGGATCTC	AAGGGCATTGCTTCGTAGATAGA
Caspase-1	CAGGCAAGCCAAATCTTTATCAC	TCCAACCCTCGGAGAAAGATG
Cytc	ACCAAATCTCCACGGTCTGTTC	ATTCTCCAAATACTCCATCAGGG
Caspase-3	GTCTGACTGGAAAGCCGAAAC	GACTGGATGAACCACGACCC
Caspase-9	GAGGTGAAGAACGACCTGACTG	CTCAATGGACACGGAGCATC
NF-kb	TCAACGCAGGACCTAAAGACAT	GCAGATAGCCAAGTTCAGGATG
TNF-α	GCCCTTCCTGTAACCAGATG	ACACGACAGCCAAGTCAACG
Caspase-8	CCGATTCTCTGGGCAACTGT	ATCCACATGTGTCCCGTTCC
GAPDH	AGAACATCATCCCAGCGT	AGCCTTCACTACCCTCTT
IL10	CGCTGTCACCGCTTCTTCA	TCCCGTTCTCATCCATCTTCTC
IL8	CGGCTTGCTAGGGGAAATGA	AGCTGACTCTGACTAGGAAACTGT
IL1β	CAGCAGCCTCAGCGAAGAG	CTGTGGTGTGCTCAGAATCCA
ZO-1	CAACGTAGTTCTGGCATTATTCG	GGGCACAGCCTCATTCCATCATCAT
Claudin-1	TGGAGGATGACCAGGTGAAG	TGTGAAAGGGTCATAGAAGG
Occludin	AGACGCGCAGTAAGATCTGG	GGGCTTCTCCGAGTAGGCAA

### Statistical Analysis

The data were processed using GraphPad Prism 5 software (GraphPad Software, San Diego, CA). Each experiment was independently conducted 3 to 4 times. Results are expressed as the mean ± standard deviation (**SD**). Group differences were assessed using a 1-way analysis of variance (**ANOVA**) with Tukey's posthoc test for multiple comparisons. *P*-value less than 0.05 was deemed to indicate the statistical significance.

## RESULTS

### Effect of LUT on the Growth and Development of Chickens Exposed to Cd

**Figure 1:**
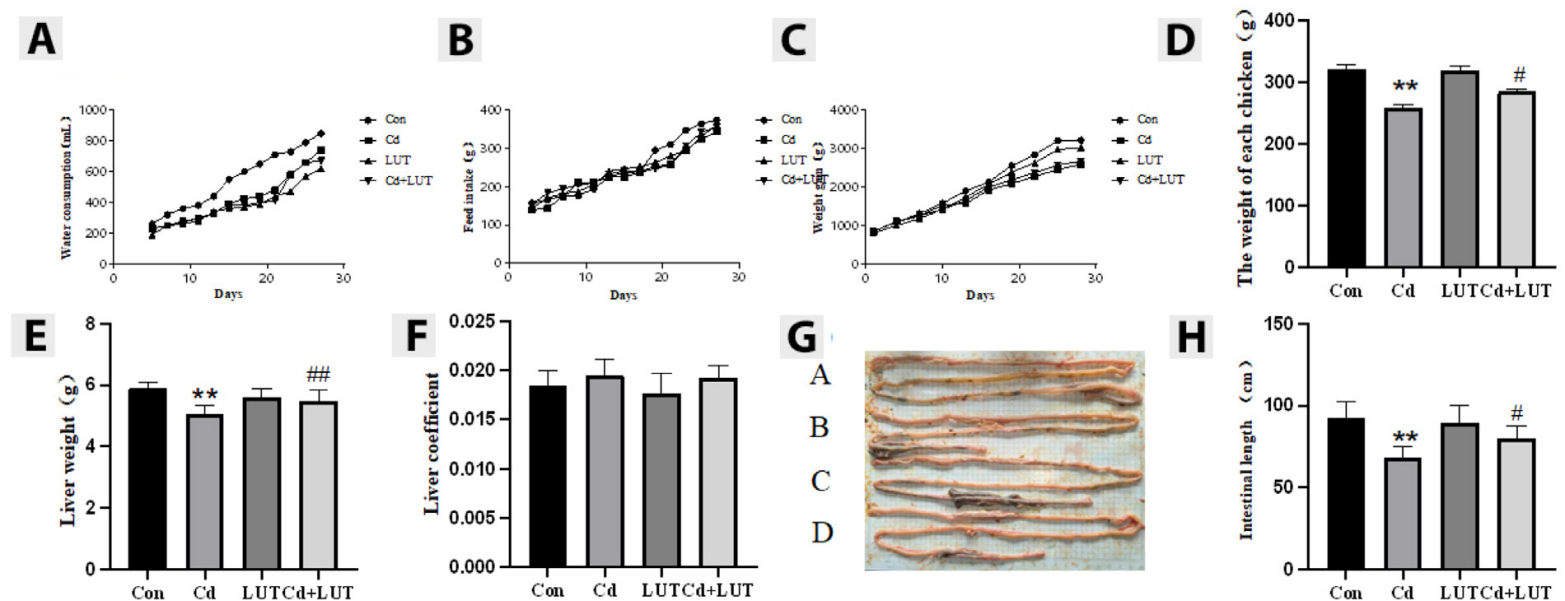
Effect of LUT on the growth and development of Cd poisoned chicken (n = 10). (A) Drinking water volume, (B) Feed intake, (C) Weight gain, (D) Total weight of chicken, (E) Liver weight, (F) Liver coefficient, (G, H) measured the length of the intestine compared with the control group, * *P* < 0.05, * * *P* < 0.01; compared with the Cd group, # *P* < 0.05 and # # *P* < 0.01.

The results showed that compared with the control group, the Cd group had a significant decreased total body weight, liver weight, and intestinal length (*P* < 0.01), while the liver coefficient had no significant change compared with the Cd group. However, LUT + Cd group showed a significant increased liver weight (*P* < 0.01), total body weight, and intestinal length in chickens (*P* < 0.05) (Figures 1A–H). The results indicate that Cd can seriously affected the healthy growth and development of chickens. Meanwhile, LUT can protect chicken growth and development.

### Effect of LUT on Liver Tissue Damage and Fibrosis Induced by Cd

**Figure 2:**
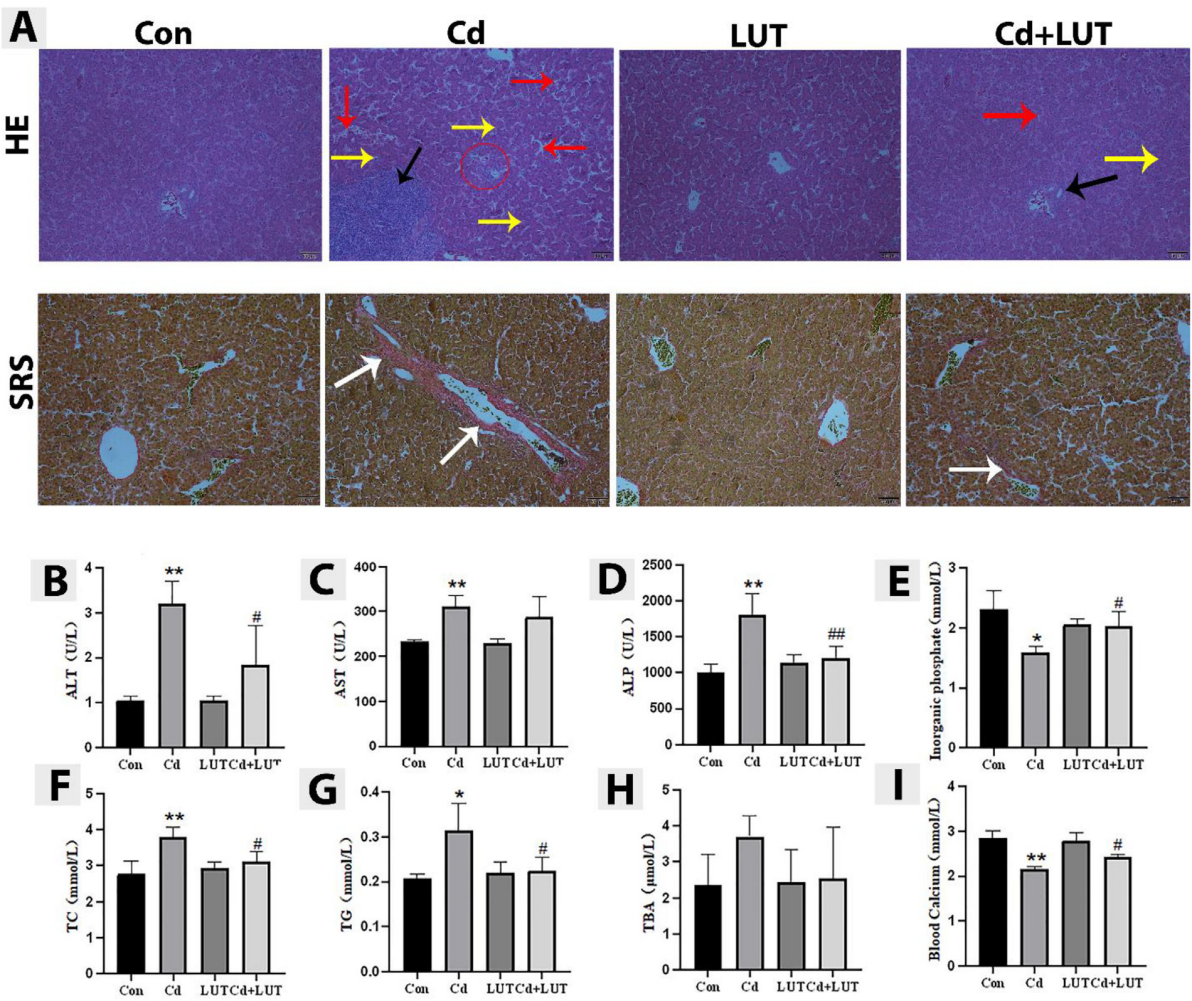
(A) HE and Sirius red staining (SRS) were used to detect the morphology and level of fibrosis in liver tissue of each group. (Scale bars 50 μm). Inflammatory cell infiltration (black arrow), nuclear shrinkage (yellow arrow), vacuolar deformation (red arrow), the red circle represents irregular arrangement of liver cell cords, and collagen fibers (white arrow). (B–I) Effects of LUT on serum biochemical markers in Cd poisoned chickens (n = 4). ALT activity, AST activity, ALP, inorganic phosphate, total cholesterol, triglycerides, serum total bile acids, and blood calcium. Compared with the control group, *P < 0.05, **P < 0.01; compared with the Cd group, #*P* < 0.05 and ##*P* < 0.01.

This study performed Hematoxylin Eosin (**HE**) and Sirius Red staining (**SRS**) on pathological tissue sections of chicken liver. The results showed that the liver tissue structure of the control group and LUT group was normal, liver lobules were clearly visible, liver cell cords were arranged regularly, liver sinus structure was clear, and liver fibrosis was almost invisible. However, the Cd group suffered from severe liver cell damage, with disordered liver lobular structure, unclear boundaries, irregular arrangement of liver cell cords, significant swelling of liver cells, increased vacuolar degeneration of liver cells, increased Kupffer cells, increased nuclear shrinkage and dissolution, and a large amount of inflammatory cell infiltration in the connective tissue of the portal vein area, resulting in severe liver fibrosis. Compared with the Cd group, the LUT + Cd group showed relief in liver cell damage, reduced inflammatory cell infiltration, and weakened liver fibrosis (Figure 2A).

Further, this study detected the activity of 3 enzymes (ALT, AST, and ALP) reflecting liver function in serum, as well as the content of inorganic phosphates, total cholesterol, triglycerides, serum total bile acids, and blood calcium. As shown in (Figures 2B-I), in the serum of the Cd group, compared with the control group, the activities of ALT, AST, ALP, and TC contents were significantly increased (*P* < 0.01), TG content was significantly increased (*P* < 0.05), blood calcium content was significantly reduced (*P* < 0.01), inorganic phosphate content was significantly reduced (*P* < 0.05), and serum total bile acid content showed no significant changes; Compared with the Cd group, the LUT + Cd group showed a significant decrease in serum ALP activity (*P* < 0.01), a significant decrease in ALT activity, TC and TG content (*P* < 0.05), and a significant increase in inorganic phosphate and blood calcium content (*P* < 0.05). There was no significant change in AST activity and serum total bile acid content.

### Effect of LUT on Intestinal Tissue Damage Induced by Cd

**Figure 3:**
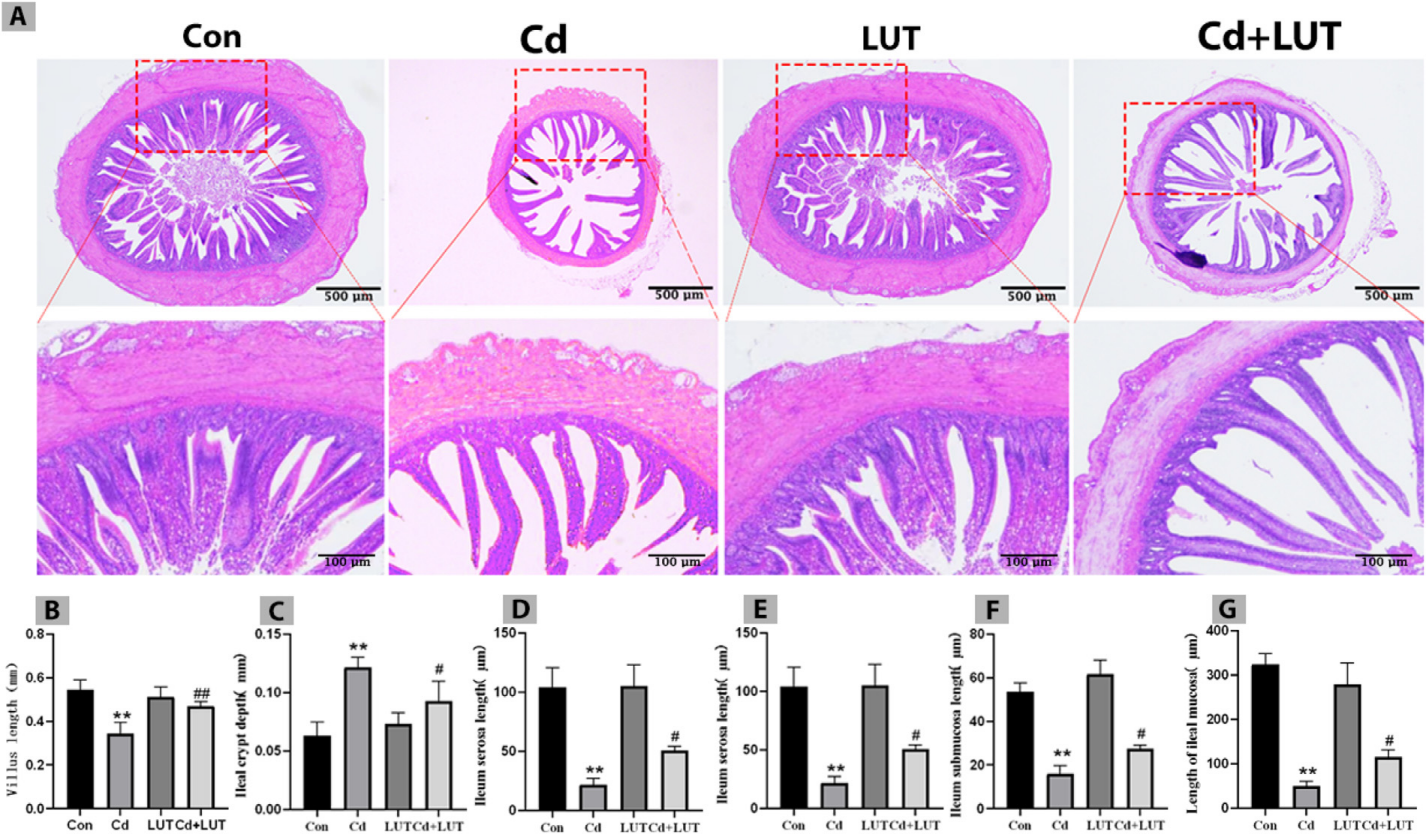
(A) HE staining was used to detect the morphology of intestine of each group. Effect of LUT on intestinal tissue damage in Cd exposed chickens (n = 4). (B) The length of small intestinal villi, (C) Depth of small intestinal crypt, (D) The thickness of the small intestine serosa layer, (E) muscle layer, (F) submucosal layer, (G) mucosal layer. (Scale bars 500 and 100 μm).

Compared with the control group, the depth of crypts in the Cd group was significantly increased (*P* < 0.01), while the length of intestinal villi and thickness of serosal layer, muscular layer, submucosal layer, and mucosal layer was significantly reduced (*P* < 0.01); Compared with the Cd group, the LUT + Cd group showed a significant increase in the length of small intestinal villi (*P* < 0.01), as well as a significant increase in the thickness of the serosal layer, muscular layer, submucosal layer, and mucosal layer (*P* < 0.05), and a significant decrease in crypt depth (*P* < 0.05) (Figures 3A–G).

### Effect of LUT on Cd Induced Liver and Intestinal Related Trace Elements and Oxidative Stress

**Figure 4:**
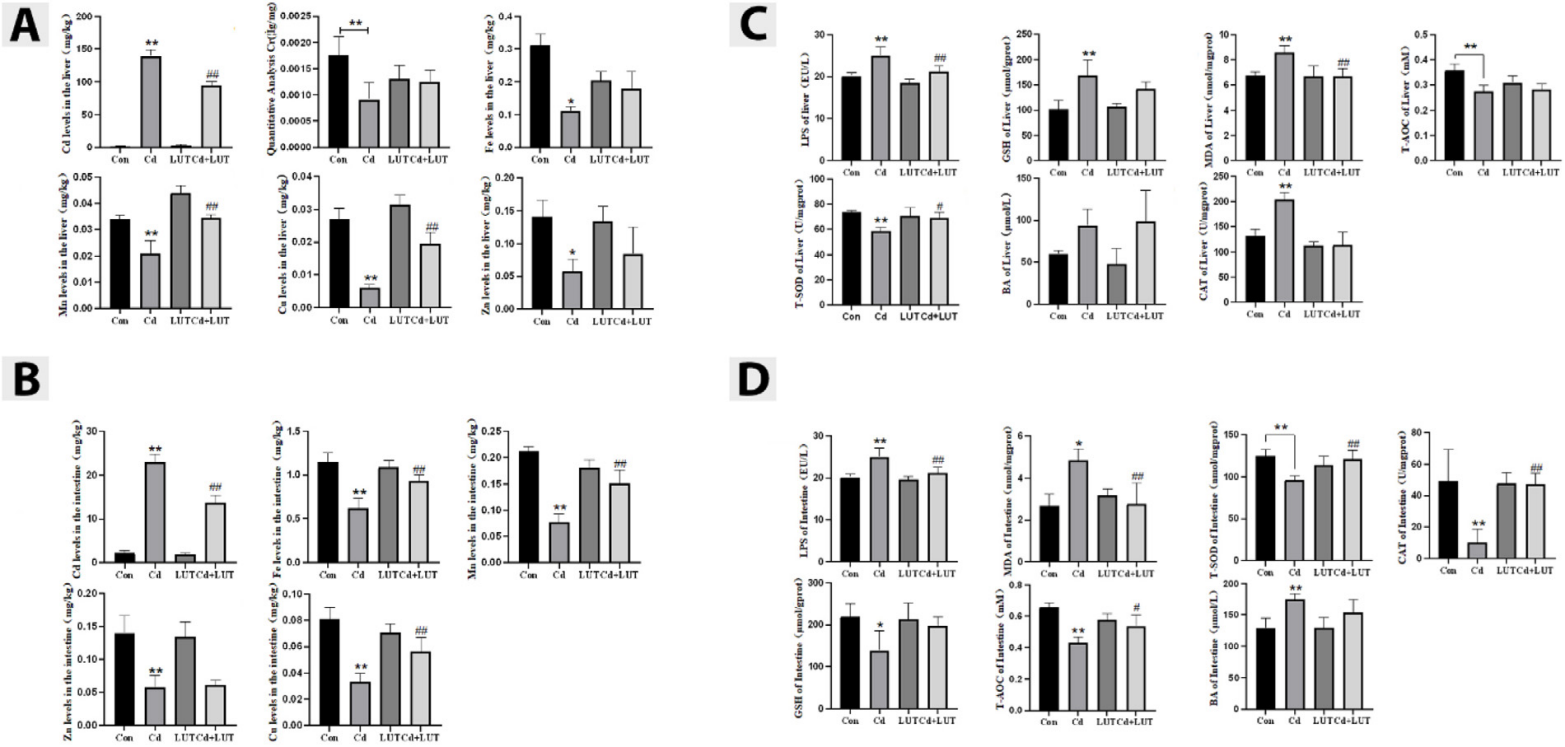
Trace element contents in the liver (n = 4). The content of Cd, chromium, iron, manganese, copper, and zinc in the liver. Compared with the control group, *P < 0.05, **P < 0.01; compared with the Cd group, #*P* < 0.05 and ##*P* < 0.01 (A). Trace element content in the intestine (n = 4). The content of cadmium, iron, manganese, zinc, and copper in the intestine. Compared with the control group, *P < 0.05, **P < 0.01; Compared with the Cd group, #*P* < 0.05 and ##*P* < 0.01. (B) Oxidative stress indicators in the liver (n = 4). (C) Liver LPS, GSH, MDA, T-AOC, T-SOD, BA, CAT. Compared with the control group, *P < 0.05, **P < 0.01; Compared with the Cd group, #*P* < 0.05 and ##*P* < 0.01. Oxidative stress indicators in the intestine (n = 4). (D) Intestinal LPS, MDA, T-SOD, CAT, GSH, T-AOC, BA. Compared with the control group, *P < 0.05, **P < 0.01; Compared with the Cd group, #*P* < 0.05 and ##*P* < 0.01.

This study detected the contents of chromium, iron, manganese, copper, and zinc elements in liver and intestine tissues. The results showed that in liver tissue, compared with the control group, the Cd group had a significant increased Cd content (*P* < 0.01), but decreased chromium, manganese, and copper contents (*P* < 0.01), and a significant decrease in iron and zinc content (*P* < 0.05); Compared with the Cd group, the LUT + Cd group showed a significant increased manganese and copper contents (*P* < 0.01), a significant decrease in Cd content (*P* < 0.01), and no significant changes in chromium, iron, and zinc contents (Figure 4A).

In the intestinal tissue, compared with the control group, the Cd group showed a significant increase in Cd content (*P* < 0.01), while the iron, manganese, copper, and zinc contents were significantly reduced (*P* < 0.01); Compared with the Cd group, the LUT + Cd group showed a significant increase in iron, manganese, and copper content (*P* < 0.01), a significant decrease in Cd content (*P* < 0.01), and no significant change in zinc content (Figure 4B).

In addition, the results showed that in liver tissue, compared with the control group, the Cd group showed a significant increased GSH concentration, MDA level, CAT activity, and LPS contents (*P* < 0.01), while T-AOC and T-SOD activities were significantly reduced (P < 0.01), and there was no significant change in BA content; Compared with the Cd group, the LUT + Cd group showed a significant decrease in LPS content and MDA level (*P* < 0.01), while there were no significant changes in GSH concentration, T-AOC, BA content, CAT activity, and T-SOD activity (Figure 4C).

In the intestinal tissue, compared with the control group, the Cd group showed a significant increase in LPS and BA content (*P* < 0.01), a significant increase in MDA level (*P* < 0.05), a significant decrease in T-AOC, T-SOD activity, CAT activity (*P* < 0.01), and a significant decrease in GSH concentration (*P* < 0.05); Compared with the Cd group, the LUT + Cd group showed a significant decrease in LPS content and MDA level (*P* < 0.01), while CAT activity and T-SOD activity were significantly increased (*P* < 0.01). There was no significant change in GSH concentration and BA content (Figure 4D).

### Effect of LUT on Cd Induced Inflammation Related Indicators

**Figure 5:**
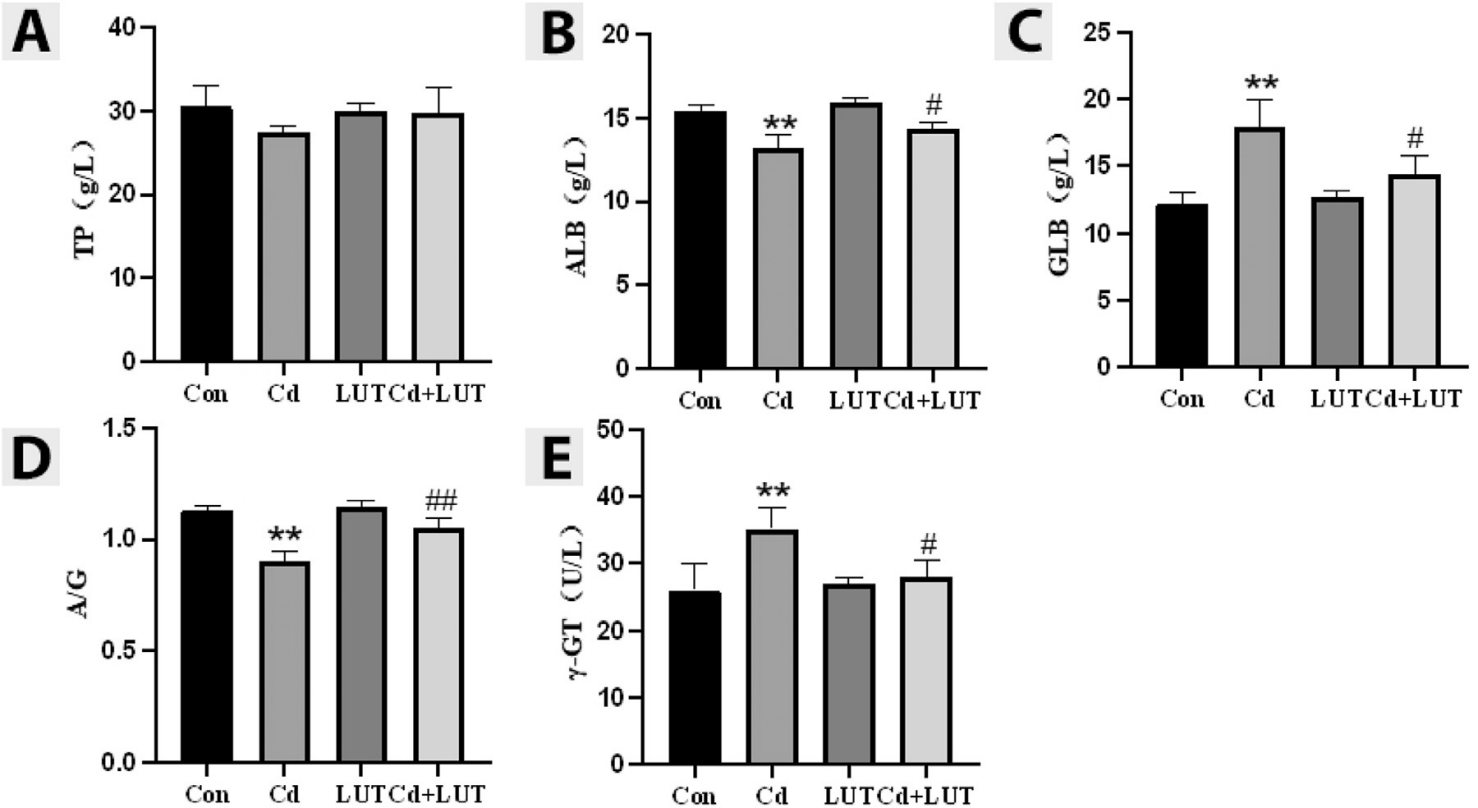
Effect of LUT on inflammation related indicators in serum biochemical markers of Cd poisoned chickens (n = 4). Measure the content of TP, ALB, and GLB, as well as the proportion of ALB/GLB, using a fully automated blood biochemistry analyzer γ-GT (A–E). Compared with the control group, *P < 0.05, **P < 0.01; Compared with the Cd group, #*P* < 0.05 and ##*P* < 0.01.

The results are shown in (Figures 5A–E), indicated that in the serum of the Cd group, compared with the control group, γ-GT activity and GLB content significantly increased (*P* < 0.01), while ALB content and A/G content significantly decreased (*P* < 0.01), and TP content showed no significant change. However, compared with the Cd group, the LUT + Cd group showed a significant increase in serum A/G (*P* < 0.01), a significant increase in ALB content (*P* < 0.05), and a significant increase in GLB content γ- GT activity significantly decreased (*P* < 0.05), while TP content showed no significant change. The results indicate that Cd-induced inflammation in chickens, and LUT can play a certain protective role against Cd.

### Effect of LUT on Cd Induced Inflammatory Damage in Chicken Liver

**Figure 6:**
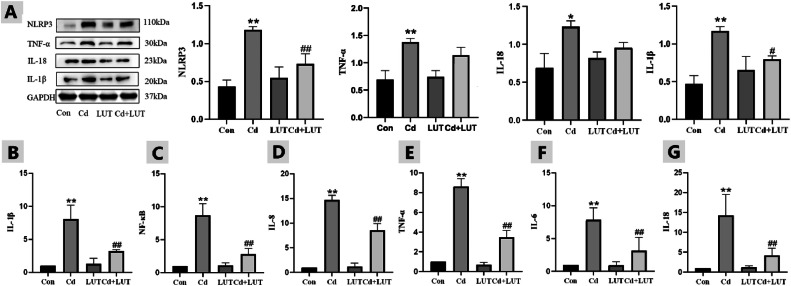
(A) Effect of LUT on the liver inflammatory response proteins in Cd exposed chickens (n=3). Compared with the control group, * *P*<0.05, * * *P*<0.01; Compared with the Cd group, # *P*<0.05 and # # *P*<0.01. Effect of LUT on the mRNA expression of inflammatory factors in Cd exposed chickens (n=3). IL-1β, NF- κB, IL-8, TNF- α, IL-6, IL-18 (B-G). Compared with the control group, * *P*<0.05, * * *P*<0.01; Compared with the Cd group, # *P*<0.05 and # # *P*<0.01.

Compared with the control group, the Cd group showed the protein expression of NLRP3, IL-18 and IL-1β was significantly increased (*P* < 0.01). However, compared with the Cd group, in the LUT + Cd group the protein expression of NLRP3, IL-18 and IL-1β was significantly reduced. Further, inflammatory related genes expression of IL-1β, NF- κβ, IL-8, TNF-α, IL-6 and IL-18 were detected by qPCR. The results showed that compared with the control group, the Cd group significantly increased gene expression of IL-1β, NF- κβ, IL-8, TNF-α, IL-6 and IL-18. However, compared with the Cd group, the LUT + Cd group showed significantly reduced genes expression of IL-1β, NF- κβ, IL-8, TNF-α, IL-6 and IL-18 (Figures 6A–G).

### Effect of LUT on Cd Induced Disruption in Intestinal Barrier of the Chicken

**Figure 7:**
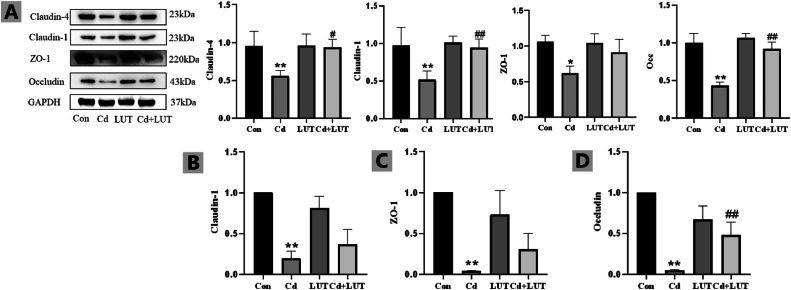
(A) Intestinal barrier protein indicators in the intestine (n = 3). Compared with the control group, *P < 0.05, **P<0.01; Compared with the Cd group, #*P* < 0.05 and ##*P* < 0.01. Effect of LUT on intestinal barrier proteins in Cd exposed chickens (n = 3). Effect of LUT on the mRNA expression of Claudin-1, ZO-1 and Occludin (B–D). Compared with the control group, *P < 0.05, **P < 0.01; Compared with the Cd group, #*P* < 0.05 and ##*P* < 0.01.

Compared with the control group, the protein expression of Occludin, Claudin-1, and Claudin-4 was significantly reduced in the Cd group (*P* < 0.01). However, compared with the Cd group, the LUT + Cd group showed significantly increased above intestinal barrier proteins. Moreover, qPCR was used to detect the gene expression of ZO-1, Occludin, and Claudin-1 intestinal barrier factors. Compared with the control group, the gene expression of ZO-1, Occludin, and Claudin-1 barrier factors was significantly reduced in the Cd group (*P* < 0.01); However, compared with the Cd group, LUT + Cd group showed significantly increased above intestinal barrier genes (Figures 7A–D).

### Effects of LUT on the Correlation Between Liver Oxidative Stress and Inflammation, Intestinal Oxidative Stress and Barrier Function in Cd Exposed Chickens

**Figure 8:**
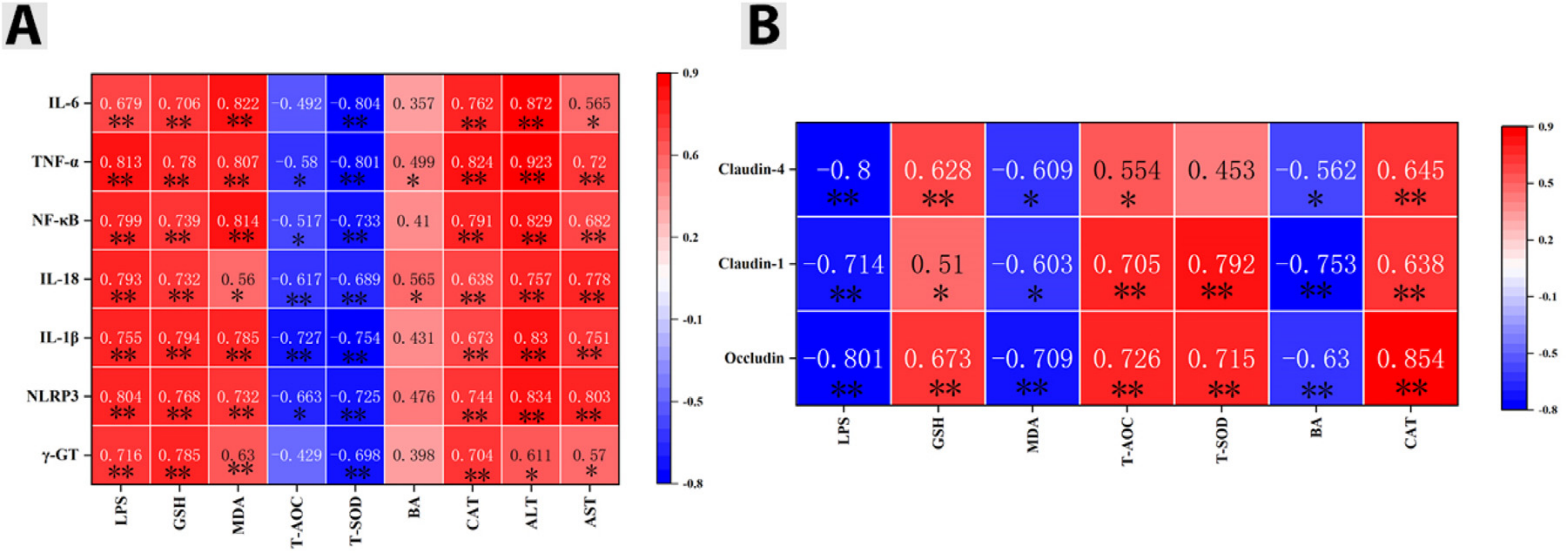
(A) Correlation between liver oxidative stress and inflammation, (B) Correlation between intestinal oxidative stress and barrier function. "*" and "* *" respectively indicate significant (*P* < 0.05) or extremely significant (*P* < 0.01) correlation between the 2 indicators.

To further investigate the effects of LUT on the correlation between oxidative stress and inflammation in Cd exposed chicken liver, as well as the correlation between intestinal oxidative stress and barrier function, this study analyzed the liver oxidative stress indicators LPS, GSH, T-AOC, BA, CAT, T-SOD, MDA, and liver inflammation indicator IL-1 using Origin software β, NF- κ B. IL-8, TNF-α, IL-6, IL-18, NLRP3 γ- The correlation of GT; The correlation between liver oxidative stress indicators LPS, GSH, T-AOC, BA, CAT, T-SOD, MDA and intestinal barrier proteins ZO-1, Occludin, Claudin-1, and Claudin-4. The results are shown in (Figures 8A–B). The results showed that in the heatmap of the correlation between liver oxidative stress and inflammatory indicators, in the T-AOC column, only IL-6 γ- GT has no correlation with it; in column BA, only TNF- α, IL-18 is correlated with it. All other data are correlated (*P* < 0.05) or (*P* < 0.01). In the heatmap of the correlation between intestinal oxidative stress and barrier function, only T-SOD was not correlated with Claudin-4, while all other data were correlated (*P* < 0.05) or (*P* < 0.01). The results showed that there was a strong correlation between Cd exposed chicken liver oxidative stress and inflammation, and a strong correlation between intestinal oxidative stress and barrier function.

### The Effect of LUT on the Diversity of Gut Microbiota in Cd Poisoned Chickens

**Figure 9:**
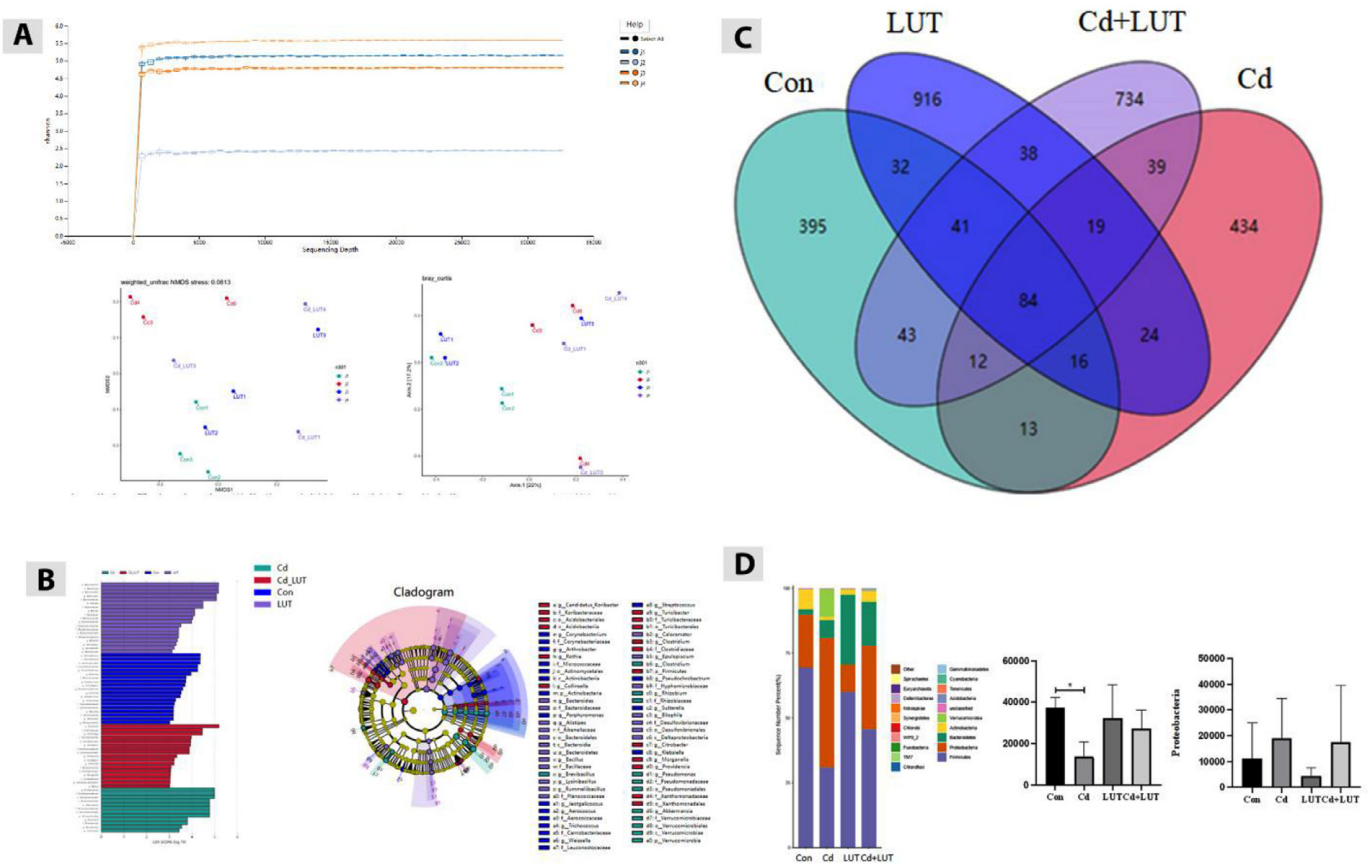
Effect of LUT on the gut microbiota of cadmium exposed chickens, the impact of changes in diversity. (A) NMDS clustering analysis of samples from different groups, and PCoA analysis of samples from different groups. (B) Distribution map of LDA values (richness) of dominant bacterial communities in different groups of samples analyzed by LEfSe. Evolutionary branching diagram of microbial communities in different groups of samples. Note: The LDA value distribution bar chart shows the species with LDA Score set to 1. (C) Venn diagram (n = 3) of the composition of various microbial communities in the colon of grass chickens. (D) The composition and relative abundance of dominant microbial communities at the phylum and order levels in the colon of grass chickens. Characteristics of gut microbiota at the phylum level in different groups of species (top 10). Firmicutes. Characteristics of gut microbiota at the phylum level in different groups of species (top 10). Lactobacillus order. Compared with the control group, *P < 0.05, **P < 0.01; Compared with the Cd group, #*P* < 0.05 and ##*P* < 0.01.

In order to understand the protective effect of LUT on Cd induced reduction of gut microbiota diversity in chickens, this study detected the gut microbiota α and β Diversity, linear discriminant analysis effect size (**LEfSe**) analysis, and branching diagram of microbial community evolution. The results are shown in (Figures 9A–B). The results indicate that in the α in terms of diversity, the Shannon index of the Cd group decreased compared to the control group; The Shannon index increased in the LUT + Cd group compared to the Cd group. Stay β In terms of diversity, both NMDS clustering analysis and PCoA analysis showed that the distance between the Cd group and the blank control group, the LUT treatment group, and the LUT + Cd co-treatment group was farther. In the LEfSe analysis, compared with the control group, there were 9 dominant bacterial groups in the control group with LDA values greater than 1, and 11 dominant bacterial groups in the Cd group with LDA values greater than 1. Compared with the Cd group, there are 3 dominant bacterial groups in the LUT + Cd group with LDA values greater than 1, and 11 dominant bacterial groups in the Cd group with LDA values greater than 1. In the evolutionary branching diagram of the microbial community, we found that the green Cd group has the smallest fan-shaped area and the shortest branching; and the other 3 groups are all relatively concentrated fan-shaped areas, with intersections and longer branches in each area, indicating that there are no significant faults in biological classification. The results indicate that Cd can cause disruption of the gut microbiota in chickens, and the addition of LUT can play a certain protective role.

### Effects of LUT on Cd Induced Changes in the Composition and Relative Abundance of Dominant Gut Microbiota

In order to explore the effects of Cd on the composition and relative abundance of dominant gut microbiota, this study analyzed the changes in microbiota between different groups through Venn diagrams and biological taxonomy. The results are shown in (Figures 9C–D). The results showed that at the phylum level, the relative abundance of Firmicutes in the Cd group was significantly reduced compared to the control group (*P* < 0.05). Compared with the Cd group, the relative abundance of Firmicutes in the LUT + Cd group showed an upward trend without significant changes. At the target level, the relative abundance of Lactobacillus in the Cd group was significantly reduced compared to the control group (*P* < 0.05). Compared with the Cd group, the relative abundance of Lactobacillus in the LUT + Cd group showed an upward trend without significant changes. The above results indicate that Cd can alter the composition and relative abundance of dominant bacterial communities in chicken intestines, but the protective effect of LUT is not very significant.

### The Effects of LUT on the Correlation Between Gut Microbiota and Liver Oxidative Stress, and Correlation Between Gut Microbiota and Barrier Function in Cd Exposed Chickens

**Figure 10:**
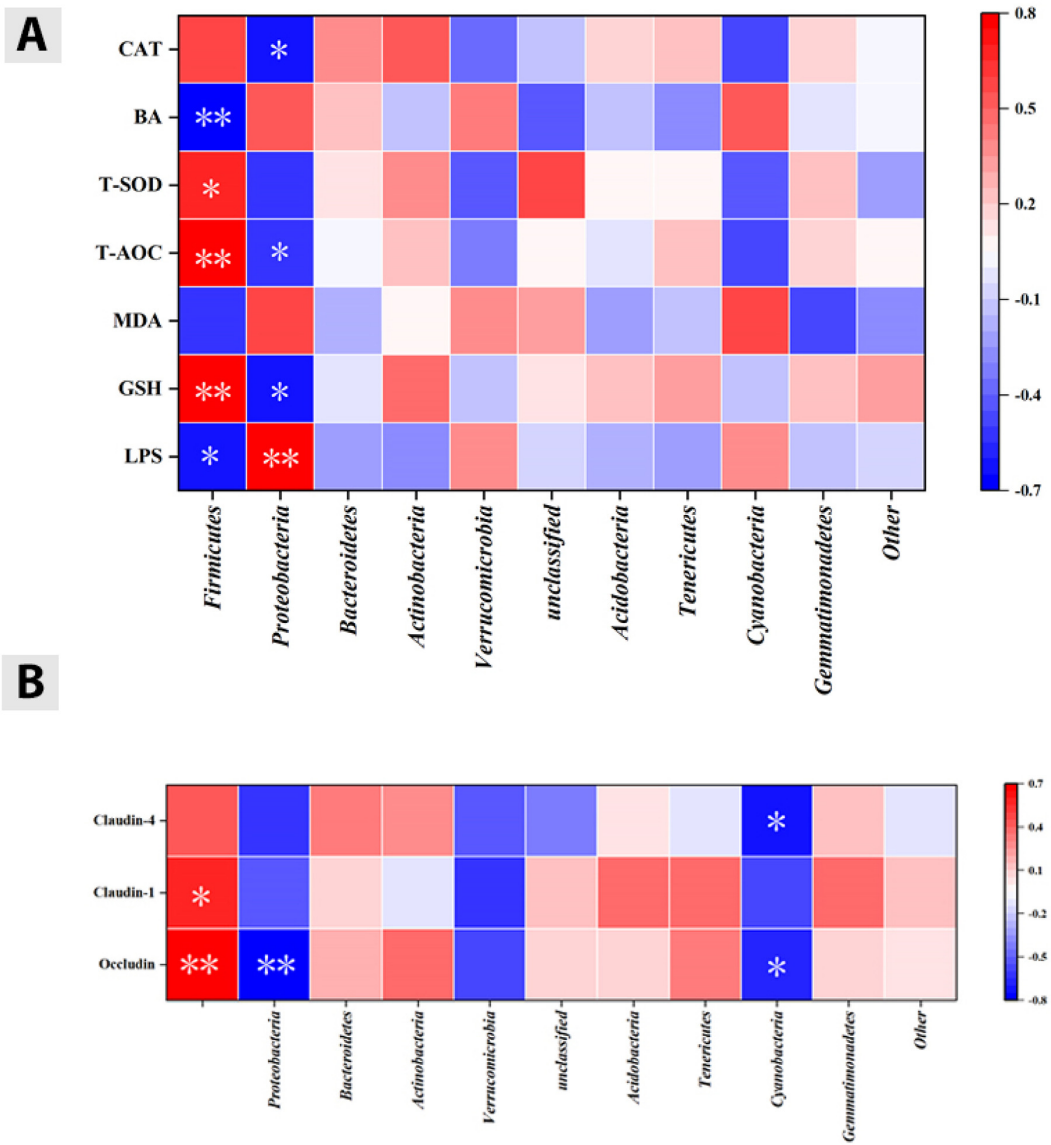
(A) Correlation between gut microbiota and liver oxidative stress. "*" and "**" respectively indicate significant (*P* < 0.05) or extremely significant (*P* < 0.01) correlation between the 2 indicators. (B) Correlation between intestinal barrier function. "*" and "* *" respectively indicate significant (*P* < 0.05) or extremely significant (*P* < 0.01) correlation between the 2 indicators.

In order to investigate the effects of LUT on the correlation between liver oxidative stress and gut microbiota barrier function in Cd exposed chickens, this study analyzed the correlation between the top 10 gut microbiota and liver oxidative stress indicators LPS, GSH, T-AOC, BA, CAT, T-SOD, MDA using Origin software; The correlation between the top 10 gut microbiota and intestinal barrier proteins ZO-1, Occludin, Claudin-1, and Claudin-4. As shown in (Figures 10A–B), under oxidative stress, LPS was negatively correlated with Firmicutes (*P* < 0.05), positively correlated with Proteobacteria (P < 0.01), GSH was positively correlated with Firmicutes (*P* < 0.01), negatively correlated with Proteobacteria (*P* < 0.05), T-AOC was positively correlated with Firmicutes (*P* < 0.01), negatively correlated with Proteobacteria (*P* < 0.05), T-SOD was positively correlated with Firmicutes (*P* < 0.05), BA was negatively correlated with Firmicutes (*P* < 0.01), and CAT was negatively correlated with Proteobacteria (*P* < 0.05).). In the function of the intestinal barrier, the intestinal barrier protein Occludin is positively correlated with Firmicutes (*P* < 0.01), negatively correlated with Proteobacteria (*P* < 0.01), and negatively correlated with Chlorophyta (*P* < 0.05). The intestinal barrier protein Claudin-1 is positively correlated with Firmicutes (*P* < 0.01), and the intestinal barrier protein Claudin-4 is negatively correlated with Chlorophyta (*P* < 0.05). The results indicate that changes in gut microbiota are correlated with liver oxidative stress and intestinal barrier function.

## DISCUSSION

Environmental pollution poses a significant challenge to the poultry industry, resulting in substantial losses and adverse effects on the health, production, and performance of avian species. It disrupts various physiological functions in birds (Hu et al., 2017).

At present, cadmium (Cd) pollution is widespread in China (Ali et al., 2022; Zheng et al., 2021). The liver is one of the main target organs for Cd-induced damage to the body. Cd can cause liver tissue damage and liver dysfunction. The most commonly used indicators for detecting liver dysfunction in clinical practice include elevated levels of ALT and AST activity in serum (Sun et al., 2023). Meanwhile, an increase in ALP activity also indicates abnormal liver function. Research has found that Cd intake can cause unclear liver tissue structure, punctate bleeding, uneven arrangement of liver cell cords, and increased liver cell swelling and vacuolization in broiler chickens (Yu et al., 2021). In a study on Cd treatment in rats, both liver weight and liver coefficient decreased, affecting normal liver function (Zou et al., 2020). Consequently, it is important to develop safe and effective medications. Luteolin (**LUT**) is a powerful natural flavonoid drug, and studies have found that it can significantly alleviate Cd-induced apoptosis and decreased vitality of human lung epithelial Beas-2B cells (Chu et al., 2021). This experiment used 150 mg/kg Lut and 100 mg/L Cd Cl_2_ to co treat Su poultry green shell grass chickens for 4 weeks. The study found that Cd can cause a decrease in total body weight, liver weight, intestinal length, blood calcium and inorganic phosphate content, while ALT, AST, ALP activities, TC and TG content increase. However, liver coefficient and serum total bile acid content did not show significant changes; the addition of LUT can increase the total body weight, liver weight, intestinal length, inorganic phosphate and blood calcium content of chickens, while reducing the activity of ALT and ALP, as well as TC and TG content. There is no significant change in the total serum bile acid content. Research has shown that the gold standard for Cd induced liver injury is a significant increase in serum ALT, AST, and ALP activity (Niture et al., 2021). At the same time, the Cd group had severe liver cell damage, disordered liver lobular structure, unclear boundaries, irregular arrangement of liver cell cords, significant swelling of liver cells, increased vacuolar degeneration of liver cells, increased Kupffer cells, and increased nuclear shrinkage and dissolution, and a large amount of inflammatory cell infiltration in the connective tissue of the portal vein area. Sirius red staining revealed severe liver fibrosis. Compared with the Cd group, the co-treatment group showed relief in liver cell damage, reduced inflammatory cell infiltration, and weakened liver fibrosis. In a study on male C57BL/6 mice, liver slices were stained with Sirius Red and it was concluded that naringenin, which is also a flavonoid drug, can alleviate liver fibrosis (Hua, et al., 2021). The depth of crypts in the Cd group increased, while the length of intestinal villi, thickness of serosal layer, muscle layer, submucosal layer, and mucosal layer thickness decreased. The addition of LUT increases the length of small intestinal villi and the thickness of the 4 layers of intestinal wall, while reducing the depth of crypts. These results indicate that this experiment not only successfully established a chicken Cd poisoning model, but also demonstrated that 150 mg/kg of LUT has no damage to the chicken body and can alleviate 100 mg/L of Cd induced and chicken body damage.

Cd can reduce mitochondrial membrane potential (**MMP**), thereby reducing mitochondrial ATP synthesis and ultimately affecting the body's life activities (Wang et al., 2014). A study has found that after Cd induction, the double layered structure of the nucleus is disrupted, and there are various adverse forms of the nucleus, such as nuclear deformation and nuclear condensation. The number of mitochondria sharply decreases and there are swelling and damage to normal structures (Zou et al., 2020). In this study, we observed severe damage to the ultrastructure of liver cells in the Cd group through transmission electron microscopy, including nuclear condensation, cytoplasmic vacuolization, and disruption of the cell bilayer membrane structure. Mitochondrial cristae are blurry and even appear vacuolized. The nucleus of the co treated group was relatively intact, the cytoplasm was relatively uniform, and the situation of mitochondria was slightly better than that of the Cd group.

Trace elements such as iron (**Fe**), manganese (**Mn**), zinc (**Zn**), and copper (**Cu**) are crucial for maintaining the body's oxidative-antioxidant balance (Mezzaroba et al., 2019). This study found that Cd exposure significantly decreased the levels of Fe, Mn, Zn, and Cu, which are essential components of the SOD coenzyme (Valko et al., 2005). In the liver, the co-treatment group showed increased Mn and Cu levels, decreased Cd levels, and unchanged Cr, Fe, and Zn levels compared to the Cd group. In the intestines, the co-treatment group had increased Fe, Mn, and Cu levels, decreased Cd levels, and unchanged Zn levels compared to the Cd group.

Research indicates that luteolin (**LUT**) can reduce malondialdehyde (**MDA**) levels, increase reduced glutathione (**GSH**) levels, enhance antioxidant enzyme activity, and boost the expression of the key antioxidant gene Nrf2 in methotrexate (**MTX**) induced rats (Dar et al., 2021). In this study, Cd exposure in liver tissue led to increased GSH concentration and catalase (**CAT**) activity, suggesting an antioxidant defense response. However, MDA levels and lipopolysaccharide (**LPS**) content, while total antioxidant capacity (**T-AOC**) and total superoxide dismutase (**T-SOD**) activities decreased. In the co-treatment group, LPS content and MDA levels decreased, with no significant changes in GSH concentration, T-AOC, CAT activity, and T-SOD activity compared to the Cd group. In intestinal tissue, the Cd group exhibited increased LPS content and MDA levels, with decreased T-AOC, T-SOD activity, CAT activity, and GSH concentration. The co-treatment group showed decreased LPS content and MDA levels, increased CAT and T-SOD activities, and no significant change in GSH content compared to the Cd group.

During the metabolic process of gut microbiota, important metabolites such as bile acids can be produced. However, if bile acids are excessive, it can activate the body's immune system, induce oxidative stress, and then change the structure of the gut microbiota through a series of pathways, forming a vicious cycle (Guo et al., 2022). A study has found that in patients with autoimmune hepatitis, the relative abundance of Clostridium, Bacteroidetes, Lactobacillus, Bifidobacterium, and Eubacterium genera increases, which leads to an increase in the content of bile acids in the body (Elsherbiny et al., 2020). In another hepatitis mouse model, the relative abundance of Clostridium in the intestine decreased, and the content of secondary bile acids also decreased, ultimately alleviating liver damage (Biagioli et al., 2019). In this study, the bile acid content in the Cd group of intestinal tissue significantly increased (*P* < 0.01), while there was no significant difference in the bile acid content in serum and liver.

LUT reduces the inflammatory markers such as γ-Glutamyltransferase (**γ-GT**) in Balb/C mice with colon cancer (Naso et al., 2016). In this study, the Cd group exhibited increased serum γ-GT activity and globulin (**GLB**) levels, decreased albumin (**ALB**) and albumin/globulin (**A/G**) ratio, with no significant change in total protein (**TP**) content. In contrast, the co-treatment group showed increased serum A/G ratio and ALB levels, decreased γ-GT activity, and unchanged TP content. These opposite changes in ALB and GLB content suggest that Cd induces inflammation in chickens, while LUT provides a protective effect.

Cd can activate the NLRP3 inflammasome, promoting liver inflammation and hepatitis, particularly severe during early estrus, by increasing TNF-α, MCP-1, and pro-inflammatory cytokines such as IL-1α, IL-1β, and IL-18 (Li et al., 2021). In chickens, Cd-induced liver injury is associated with elevated mRNA and protein levels of inflammatory factors like iNOS, NF-κB, TNF-α, and PTGE (Wang et al., 2020). This study found that Cd exposure increased NLRP3, IL-1β, and IL-18 protein expression, as well as the expression of IL-1β, NF-κB, IL-8, TNF-α, IL-6, and IL-18 genes. The co-treatment with LUT significantly reduced NLRP3 and IL-1β protein levels and decreased the expression of IL-1β, NF-κB, IL-8, TNF-α, IL-6, and IL-18 genes, though IL-18 protein levels remained unchanged. These results indicate that LUT has a protective effect against Cd-induced liver inflammation in chickens.

Cd damages the intestinal barrier, leading to a significant loss of goblet cells and increased vulnerability to pathogens, exacerbating inflammatory bowel disease (**IBD**). Inhibiting reactive oxygen species (**ROS**) production and the Notch pathway can enhance the intestinal barrier and preserve goblet cells (Xie et al., 2020). Cd exposure compromises intestinal integrity, heightens the risk of intestinal diseases, and induces liver inflammation and oxidative stress. LUT can inhibit Cd-induced ROS generation, enhancing intestinal function and microbiota, increasing barrier protein expression, reducing blood lipopolysaccharides, and inhibiting the TLR4/NF-κB inflammatory pathway, thus preventing the progression from simple steatosis (**SS**) to nonalcoholic steatohepatitis (**NASH**) (Wang et al., 2018). In this study, the Cd group showed reduced expression of Occludin, Claudin-1, and Claudin-4 proteins and genes in intestinal tissue. The co-treatment group exhibited increased expression of these proteins and genes compared to the Cd group.

## CONCLUSION

In summary, cadmium (**Cd**) exposure can disrupt gut microbiota, and the observed changes in the relative abundance of gut microbiota are consistent with Cd-induced oxidative stress damage to the liver and intestines, as well as decreased intestinal barrier function. This oxidative stress damage is strongly correlated with liver inflammation, indicating that Cd can impair liver and intestinal function in chickens. However, the addition of luteolin (**LUT**) was found to alter the structure of the gut microbiota positively. LUT increased both α diversity and β diversity of the microbiota, enhanced the relative abundance of dominant microbiota, and reduced the extent of evolutionary branching. Therefore, LUT can mitigate Cd-induced disruptions in gut microbiota, highlighting its potential as a protective agent against Cd-induced liver and intestinal damage.

## DISCLOSURES

The authors declare that they have no known competing financial interests or personal relationships that could have appeared to influence the work reported in this paper.
